# Update in Biomolecular and Genetic Bases of Bicuspid Aortopathy

**DOI:** 10.3390/ijms22115694

**Published:** 2021-05-27

**Authors:** Alejandro Junco-Vicente, Álvaro del Río-García, María Martín, Isabel Rodríguez

**Affiliations:** 1 Cardiology Department, Heart Area, Hospital Universitario Central de Asturias (HUCA), 33011 Oviedo, Spain; ajuncovicente@gmail.com; 2 Cardiac Pathology Research Group, Instituto de Investigación Sanitaria del Principado de Asturias (ISPA), 33011 Oviedo, Spain; lvaro1997@gmail.com; 3 REDinREN from Instituto de Salud Carlos III (ISCIII), 28040 Madrid, Spain

**Keywords:** aortopathy, bicuspid aortic valve, bicuspid aortopathy, biomarkers, congenital heart disease, genetics

## Abstract

Bicuspid aortic valve (BAV) associated with aortopathy is the most common congenital heart disease in the general population. Far from being a simple harmless valve malformation, it can be a complex and heterogeneous disease and a source of chronic and acute pathology (early valvular disease, aneurysm, dissection). In the previous years, intense research has been carried out to find out and understand its mechanisms, but the pathophysiology of the disease is still not fully understood and many questions remain open. Recent studies have discovered several genetic mutations involved in the development of valvular and aortic malformations, but still cannot explain more than 5–10% of cases. Other studies have also focused on molecular alterations and cellular processes (TGF-β pathway, microRNAs, degradation of the extracellular matrix, metalloproteinases, etc.), being a field in constant search and development, looking for a therapeutic target to prevent the development of the disease. Increased knowledge about this multifaceted disorder, derived from both basic and clinical research, may influence the diagnosis, follow-up, prognosis, and therapies of affected patients in the near future. This review focuses on the latest and outstanding developments on the molecular and genetic investigations of the bicuspid aortopathy.

## 1. Introduction

**Figure 1:**
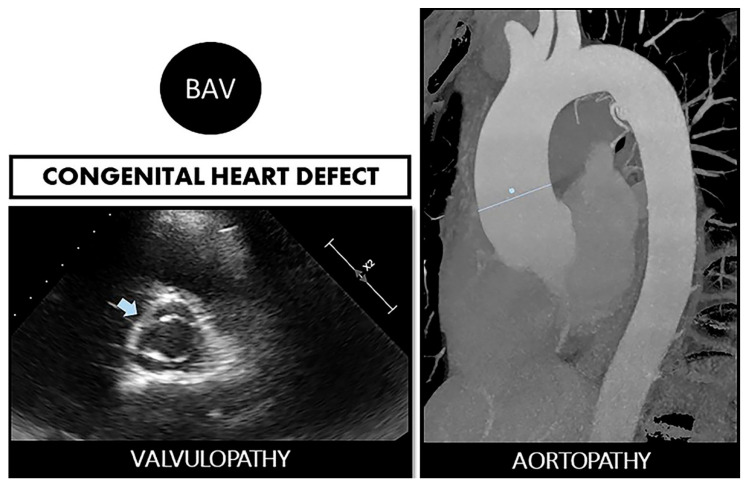
Bicuspid aortopathy is a double road. The congenital defect can develop in the person who suffers from both valvulopathy (stenosis or regurgitation) and aortopathy (aneurysms and risk of spontaneous dissection). On the left, echocardiographic image of BAV (arrow). On the right, a resonance image of dilated ascending aorta (marked straight line).

It has long been known that the bicuspid aortic valve (BAV) is the most common congenital heart defect, with a prevalence differing according to studies (0.5–2% of births) [1,2]. With many cases in young people, it generates a high annual morbidity and mortality, derived not only from valve dysfunction (stenosis due to early calcification or aortic regurgitation) but also from aortic complications (Figure 1). It has been shown, depending on the reports, that up to 35–80% of patients with BAV associate dilation of the ascending aorta [3,4]. Therefore, the patients have a higher age-adjusted relative risk of aortic dissection than the general population. Given the close relationship between valve alteration and its aortic continuity, the term “bicuspid aortopathy” has begun to be used in the literature [5].

Classically, an autosomal dominant inheritance has been described, but appears with reduced penetrance (same genetic variant but no disease) and variable expressivity (same genetic variant with different manifestations of disease) [6]. In any case, it is recognized that screening in family of the index case is necessary because the prevalence of BAV in first-degree relatives is 10 times higher than that in the general population [7].

Since only symptomatic patients seek medical attention, there is a knowledge gap about the real consequences of having BAV, and thus, many patients remain underdiagnosed. BAV is generally diagnosed in adulthood with the onset of aortic valvular dysfunction [8]. Practicable methods include both transthoracic (TTE) and transesophageal echocardiography (TEE), cardiac computed tomography (CT), and cardiac magnetic resonance (CMR).

More than 50% of patients with BAV undergo aortic valve replacement during their lifetime, and more than 25% of patients with BAV undergo aortic surgery performed for dilation of the aortic root or ascending aorta, often concurrent with aortic valve replacement [4,9].

In the last decade, many studies have been published focusing on the finding genetic and molecular alterations present in families with BAV and consistent inheritance, through registries of patients with BAV and tricuspid aortic valve (TAV), looking for differences between them [10]. Isolated mutations, several genes, cellular pathways, different microRNAs, histopathological alterations, etc. have been found associated with the bicuspid aortopathy. Many pieces of this immense puzzle are unraveled in the following pages, being aware that in the coming years, the task will consist of interconnecting these elements. Consequently, we can say that despite intense research in the recent years, the manifest genetic, epigenetic, and molecular heterogeneity and complexity of this pathology means that the final pathogenic and ontogenetic mechanisms are still not fully understood. This article reviews the latest and outstanding advances in molecular and genetic investigations on bicuspid aortopathy.

## 2. BAV and Aortopathy: A Two-Way Road

The etiology and pathogenesis that lead to dilation of the ascending aorta in patients with a BAV are uncertain to date. The actual incidence and prevalence of aortopathy are not truly known either, since it is often an alteration with an asymptomatic course. Although, as classic studies explain, aortic dilation is a relatively common complication in patients with BAV, appearing at an earlier age and progressing continuously and rather faster [11,12]. Two theories have been proposed, two non-exclusive roads.

One of the roads is the “genetic theory”. As we have explained later, this theory defends the existence of mutations in different genes in the patients who carry a double leaflet valve and that affect the aorta at the same time. This connection should not be surprising since the aortic valve and the ascending aorta share the same embryological origin [13].

The other road is the “hemodynamic theory”. Due to the anomalous opening of the BAV, an eccentric flow is formed directed against the aortic wall, this constant tangential force being the one that ends up generating the dilation. The consequent load is known as “wall shear stress” (WSS) [14].

In the past decade, using 4D flow cardiovascular magnetic resonance (CMR 4D), it was found that the flow patterns in the ascending aorta of the patients with RL-BAV (fusion subtype of the right and left coronary cusps) and RN-BAV (fusion subtype of the right and non-coronary cusps) were different: RL-BAV impacted the anterior aortic wall and RN-BAV influenced directly on its posterior side, explaining different aortic dilation morphotypes [15,16]. However, there are also studies reporting a weak or no association between the BAV morphotype and the location of the aneurysm [17,18]. To elucidate these knowledge gaps, many subsequent studies continue to emerge in the recent years in this field. However, the subtypes according to the location of the raphe affect aortic flow. Moreover, valve degeneration has been shown to affect flow and WSS, being more severe and rotational in the patients with severe stenosis independently of valve morphology [19].

It has been suggested in BAV that aortic dilation development is an adaptive mechanism of the arterial wall. A remodeling through mechano-sensing is possible. Regions with increased WSS have an extracellular matrix dysregulation and elastic fiber degeneration, with higher levels of MMP-2, MMP-3, and TGF-β in the areas with the higher WSS, associated with a greater loss of architecture in the middle layer [20,21]. It has also been observed that patients with BAV-aortic stenosis present increased WSS and thinning and loss of elastic fiber architecture more markedly than those with BAV-aortic regurgitation. In addition, the regional histopathology alteration manifests itself more strongly in mildly aortic dilation (<4.5 cm) [22,23].

However, not all patients with the same valvular phenotype have the same pattern of aortopathy and, furthermore, 25–35% of BAV present a non-dilated aorta in the follow-up [3,24]. However, controversies continue to exist. For example, first-degree relatives (with TAV) of patients with BAV suffer more frequently from aortic aneurysms, without presenting these eccentric flows involving WSS [25]. In a recent meta-analysis by Girdauskas et al., the appearance of aortic dissection on post-aortic valve replacement was evaluated in the patients with BAV. It was found that those with regurgitation had a higher risk of dissection at follow-up, which could indicate greater tissue weakness. This occurs, even with the new valve replaced, where the WSS secondary to the altered outflow is not present. In contrast, BAV stenosis-associated aortopathy seems to follow a more benign course post-aortic valve replacement [26].

Other non-hemodynamics factors must be linked. Currently, the results do not clearly indicate that hemodynamic effects on the aorta, due to outflow eccentricity, explain bicuspid aneurysms by themselves. Longer follow-up records (even decades) of patients with BAV are necessary to achieve clarity about the relationship of WSS and aneurysms, especially in non-dysfunctional BAVs.

**Figure 2:**
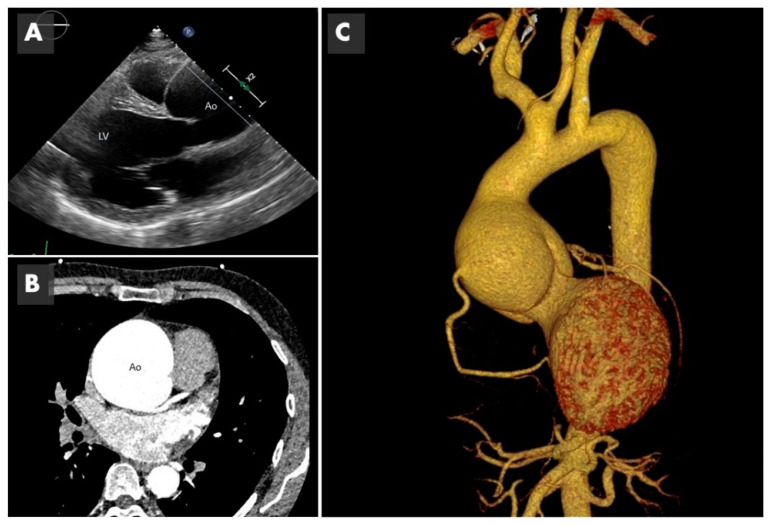
Imaging of bicuspid associated aneurysm. (**A**) The 2D image of a transthoracic echocardiogram in the parasternal long axis plane showing the dilated aorta (Ao) marked with the straight line (LV: left ventricle). (**B**) Computed tomography (CT) in axial plane showing the very dilated ascending aorta (Ao) at the level of the sinuses of Valsalva. (**C**) The 3D CT reconstruction, showing a huge 8 cm aneurysm.

It seems more sensible to think that the two phenomena (genetics + hemodynamics) participate. The impaired aortic ejection flow, with a weak arterial wall that is already genetically determined or histo-molecularly altered, will possibly cause or accelerate the development of aneurysms (Figure 2). Understanding the mechanisms underlying vascular remodeling of the aortic wall is essential for the development of new risk scales, not based on measurements of aortic size, but on imaging parameters or biomarkers, which allow establishing the best individualized surgical time.

## 3. Genetics Insights into Bicuspid Aortopathy

The BAV disease is considered nowadays as an autosomal dominant disorder with low penetrance and variable expressivity. Although different genes with divergent inheritance pattern have been associated to this entity, its genetic bases are still not completely known [27]. BAV can present as an isolated condition or associated with syndromic disorders such as Marfan [28] and Turner syndromes [29] and with other cardiovascular diseases such as coarctation of aorta and ventricular septal defect. Familial clustering was initially suggested in different studies [30,31] and the first estimation of its heritability was proposed by Cripe et al. [32]. Here, we review the genetic basis of BAV aortopathy and some of the candidate genes that have been implicated in the process (Table 1).

The main gene with the strongest evidence that has been demonstrated to be associated with BAV in both familial and sporadic forms is *NOTCH1* (9q 34.4, OMIM 190198). The NOTCH1 pathway is directly involved in the development of the valve-forming fields during cardiogenesis and is crucial for the correct development of endocardial cushions through regulation of endocardial epithelial-to-mesenchymal transition and remodeling of the immature aortic valve [33]. Mutations in *NOTCH1* cause an early developmental defect in the aortic valve and a later de-repression of calcium deposition that causes progressive aortic valve disease [34]. Besides, loss-of-function mutations in *NOTCH1* have also been associated with other congenital heart diseases involving the left ventricle outflow tract, mitral valve, and, less frequently, with right heart cardiac lesions, in an autosomal dominant trait with variable expressivity [47]. Otherwise, thoracic aortic aneurysms have also been described in pedigrees with inactivating *NOTCH1* mutations. Thus, the study published by Kerstjens-Frederikse et al., determining the prevalence and spectrum of *NOTCH1* mutations in left-sided congenital heart disease, found that thoracic aortic aneurysms occurred in six mutation carriers [48].

Furthermore, members of the GATA family of the zinc finger superfamily of transcriptions factors have also been related to BAV disease [49]. This family consists of six members divided in two subgroups based on their sequence homology and tissue location: GATA1, 2, and 3 are mainly implicated in hematopoietic development and GATA4, 5, and 6 are essential in cardiovascular embryogenesis, as well as in other tissues derived from mesoderm and endoderm. Accordingly, a *GATA4* (8p23.1, OMIM 600576), loss-of-function mutation has been associated with enhanced susceptibility to BAV [35]. *GATA5* (20q13.33, OMIM 611496) has been demonstrated to be involved in cardiac morphogenesis and aortic valve development implicated in BAV pathogenesis [37]. Our group have previously identified four genetic variants in *GATA4*, *GATA5,* and *GATA6* (18q11.2, OMIM 601656) only present in patients with BAV, with a potential pathogenic effect predicted by bioinformatic tools, supporting the implication of these genes in the development of this valvulopathy [36]. A *GATA6* loss-of-function mutation has also been associated with enhanced susceptibility to familial BAV and also recent studies suggest that haploinsufficiency of transcription factor GATA6 leads to BAV [38,39].

Another cardiac transcription factor recently associated with BAV is *TBX5* (12q24.21, OMIM 601620). This gene, previously associated with atrial fibrillation, was sequenced in unrelated adult patients suffering from both congenital heart defects and atrial fibrillation. A novel nonsense mutation that impairs the transcriptional activity of the protein was found segregating also with BAV in the family [40]. This finding highlights the importance of deciphering the molecular pathways implicated in order to identify new candidate genes for genetic studies.

The transcriptional regulator encoded by *SMAD6* gene (15q22.31, OMIM 602931) has also been confirmed to be related to BAV and associated thoracic aortic aneurysm [41]. Luyckx et al. identified seven novel likely pathogenic *SMAD6* variants in BAV individuals with thoracic aortic aneurysm, establishing the role of *SMAD6* variants in their etiology and revealing limited contribution to thoracic aneurysms development in patients with TAV. Familial segregation studies confirmed reduced penetrance and variable clinical expressivity. The results of these studies improved insights into the clinical spectrum of *SMAD6*-related BAV-thoracic aneurysms [42].

*FBN1* gene (15q21.1, OMIM 134797) encodes an extracellular glycoprotein (fibrillin-1), component of connective tissue that is secreted by vascular smooth muscle cells, and regulates the structural integrity of the aortic media. Previous studies have evidenced the presence of genetic variants in *FBN1* gene in isolated BAV patients with aortic root dilation and in Marfan syndrome patients with BAV [43]. Regarding the superior prevalence of BAV in patients with Marfan syndrome to the general population [28], common underlying mechanisms for these two entities have been proposed: increased metalloproteinase (MMP) activity and a decreased fibrillin-1 expression in the aortic wall [50]. Moreover, fibrillin-1 deficiency could be responsible for matrix alterations, which contribute to aortopathy associated with BAV without Marfan syndrome [51].

Another candidate gene is *ROBO4* (11q24.2, OMIM 607528) in which Gould et al. found two mutations segregating with ascending aortic aneurysm in BAV subjects in two families. The involvement of ROBO4 in BAV formation and in BAV-associated ascending aortic aneurysm was confirmed using animals deficient for *Robo4* as well as by endothelial cell-specific *Robo4* silencing or mutation [44].

Finally, missense mutations in *ACTA2* gene (10q23.31, OMIM 102620), encoding smooth muscle α-actin, had been shown to be responsible for 14% of familial ascending aortic aneurysms and dissections [45]. However, this gene, although candidate, does not seem to play a significant role in the pathogenesis of BAV aortopathy as concluded by Tortora et al. in a small cohort of patient [52].

Not only mutations but also polymorphisms could prove important in the early identification of the patients at higher risk. The functional polymorphism rs2071307 in the elastin gene (*ELN*, 7q11.23, OMIM 130160) has been shown to correlate with circulating elastin soluble fragments and ascending aorta diameter in BAV patients [46]. These facts could be an important starting point in the explanation of aneurysm formation in these subjects, and future studies will have to show whether increased spatial heterogeneity and temporal dispersion of aortic strain distribution might be specific signs of aneurysm formation.

As a conclusion, and as Messner et al. reflect in their recent review, although there is a large list of genes linked to BAV and BAV-aortopathy, we are still far from a complete knowledge of this complex and heterogeneous disease [10]. Many “genetic” questions still remain.

## 4. Pieces and Findings in Epigenetics

Epigenetic mechanisms play a key role in the embryonic development of the heart, and their dysregulation can be the cause of the manifestation of various pathologies [53]. To date, an aberrant epigenetic regulation have been described in several heart diseases such as cardiac hypertrophy [54], myocardial ischemia [55], and, specifically, the aortopathy of patients with BAV. In this case, it has been suggested that the altered blood outflow due to the BAV may cause a dysregulation of epigenetic mechanisms that leads to aberrant gene expression and then to the complications associated to BAV [56], complementing the hemodynamic theory of their pathogenesis [57].

Epigenetic mechanisms primarily encompass DNA methylation and histone modification. DNA methylation usually takes place in CpG dinucleotides grouped in CpG islands where it results in the stable silencing of gene expression [58]. In the case of patients with BAV aortopathy, results from Pan et al. showed a differential gene methylation profile in patients with BAV compared to those with TAV [59]. Specifically, another study observed hypomethylation and hypermethylation of the *ACTA2* and *GATA4* genes, respectively [60]. Another study reported a pattern of hypomethylation in genes related to the epithelial-to-mesenchymal transition process. This finding suggests that the alteration in the regulation of this process in the ascending aorta may be the cause of developing aortic aneurysm [61]. These reports show a relationship between patients with BAV and an aberrant DNA methylation process, however, future research is necessary to determine whether this discordant methylation process is the cause or the consequence of BAV or associated aortopathy.

Likewise, the role played by histone modification is also crucial. Modification of the histone H3 marker near the promoter of the *SMAD2* gene has been observed in patients with BAV-associated thoracic aortic aneurysm as an epigenetic mechanism behind SMAD2 overexpression [62]. This suggests a malfunction of cellular processes related to tissue repair, extracellular matrix remodeling, proliferation, and migration, due to dysregulation of the TGF-β/SMAD pathway, which plays a major role in vascular remodeling [63].

## 5. Pieces and Findings Related to MicroRNAs and Other Regulatory RNAs

Post-transcriptional mechanisms of regulation involving non-coding RNAs (ncRNAs) must also be taken into account, focusing mainly on microRNAs (miRNAs) and long non-coding RNAs (lncRNAs).

miRNAs are 22–25 nucleotide ncRNAs that negatively regulate protein synthesis by inhibiting protein translation or by promoting the cleavage of mRNAs [64]. Currently, it is known that an atypical expression of miRNAs occurs in different cases of aortopathies related to BAV [65], and this abnormal expression may be due to the presence of polymorphisms in genes that encode for these miRNAs [66].

Different authors have described miRNAs with differential expression levels in patients with BAV compared to patients with TAV (Table 2).

Yanagawa et al. reported 34 out of 1583 miRNAs with differential expression, highlighting the down-regulation of miR-141 [67]. In addition, recently, miR-3688 and miR-424 have been proposed as new molecular features, which targets important components of the Hippo and TGF-β pathways, respectively, and being the latter one deregulated in BAV patients [72].

Differences were also reported in the expression of miRNAs between BAV patients with aortic stenosis and aortic regurgitation. This difference occurred mainly in those miRNAs that negatively regulate the expression of genes related to calcification [69].

Another study also suggests that the regulation of specific miRNAs in aortic tissue, specifically miR-133a and miR-143, may affect the development of aneurysms in patients with BAV due to its effects on MMP/TIMP homeostasis [70].

As a result of all these studies, miRNAs have begun to be considered as potential biomarkers for the prediction and prognosis of different pathologies. This is observed in the case of patients with BAV in whom Martinez-Micaelo et al. reported that circulating miR-122 and miR-486 were downregulated, while miR-130a was upregulated. In addition, in patients with aortic dilation, their aortic diameter was inversely correlated with plasma levels of miR-718 [71].

lncRNAs are currently being considered as potential biomarkers of various pathologies, including cardiovascular pathologies [73]. lncRNAs are molecules longer than 200 nucleotides, and their cardiovascular regulatory functions are still poorly characterized [68]. A study carried out by Carrion et al. showed that lncRNA HOTAIR levels are decreased in BAV compared to TAV tissue and in human aortic interstitial cells exposed to cyclic stretch. In addition, they demonstrated that repression of HOTAIR in vitro led to an increase in the expression of genes related to calcification, which suggests that this mechano-response of HOTAIR is one of the reasons why there is an accelerated calcification in patients with BAV [74].

## 6. Molecular and Cellular Biology of the Aortic Valve and Associated Aortopathy

Nowadays, with the implementation of the omics techniques in research laboratories, it is being possible to discern the characteristic molecular phenotypes of different cardiovascular diseases [75]. Due to this, recent studies are directing their efforts to verify if there is a molecular phenotype associated with BAV and its associated aortopathies. The identification of some protein, signaling pathway, or some key expression pattern in BAV patients could be vital for the early diagnosis of the disease and to delay or avoid the development of valvular and aortic complications, as well as for the identification of therapeutic targets that help in the development of a treatment that can palliate and improve the condition of BAV aortopathy patients.

At first and currently thriving, many studies focused on the modifications of the NOTCH1 signaling pathway, since as previously mentioned, NOTCH1 was described as the gene with the strongest evidence to be implied in BAV development. In fact, Malashicheva et al. reviewed NOTCH signaling in aortic pathologies describing differences between TAV- and BAV-associated aortopathies. They highlight the reduced expression of genes from NOTCH pathway NOTCH1, NOTCH4, and DLL4 in the aortic endothelial cells of patients with BAV compared to cells from healthy persons. However, the expression levels of direct NOTCH target genes HEY1 and SNAI1 were not different between patients and controls [76]. Previous studies in this sense, comparing BAV and TAV, reported that individuals with BAV had significantly reduced plasma levels of NOTCH1 when compared to TAV subjects. Expression levels of NOTCH-related genes (NOTCH1-4, HEY1, and NOTCH-regulated ankyrin repeat protein) were significantly lower in both aneurysmal and normal portions of aortic tissues from subjects with BAV compared to patients with TAV [77,78]. The expression of NOTCH-related genes was also different in valve interstitial cells from patients with calcific aortic stenosis with BAV or TAV [79]. Finally, authors concluded that early signaling events including NOTCH-dependent mechanisms are responsible for the initiation of aortic valve calcification and are different between patients with BAV and TAV [79].

Another important factor related to BAV aortopathy is nitric oxide (NO). The NO secreted by vascular endothelial cells promotes the dilation of blood vessels, and changes in its signaling or bioavailability can alter vascular homeostasis and thus, become an important modulator of the development of cardiovascular diseases such as aortic aneurysm [80]. According to this, the relationship between NO signaling and BAV aortopathy has been described. In this sense, Peterson et al. used a mouse model deficient in NO synthase 3 (*Nos3*^−/−^), essential for the balance of NO, which present congenital BAV. These mice developed dissections in the ascending aorta as a result of changes in NO signaling that disturb the profile of elastin [81]. Another study, carried out by Gauer et al., showed that in non-dilated ascending aorta wall from BAV patients, there were lower levels of NOS3 than in those with TAV. However, this difference was not present in the case of dilated ascending aortas, suggesting that aberrant eNOS expression in BAV aortas may precede aneurysm development. This finding would support the theory of the genetic factors rather than hemodynamic factors as the cause of BAV [82].

Furthermore, starting with a transcriptomic analysis of ascending aorta tissue from BAV and TAV patients with and without aortic dilation, Hirata et al. concluded that AKT was activated (phosphorylated) in the BAV ascending aorta, irrespective of the aortic dilation, compared to the TAV aorta [83]. The involvement of AKT kinase in aortopathy is not completely elucidated but AKT is part of a signaling pathway that regulates cell growth and differentiation and is involved in tissue remodeling. Therefore, AKT pathway could be involved in the expansion of the aortic diameter frequently associated with BAV [83].

Finally, it is also important to highlight the results from proteomic and metabolomic studies of BAV aortopathy. In the work of Skeffington et al., the proteome profile in aorta samples of young children with coarctation of the aorta differed between patients with BAV and TAV, with a greater presence of proteins related to the inflammatory acute phase response being observed in BAV patients. Similarly, a higher protein profile corresponding to signaling pathways related to apoptosis and oxidative stress was also observed [84].

Another proteomic study showed a different protein profile in BAV compared to TAV just in the convexity, known to be exposed to different hemodynamic stimuli, and not in the concavity of the ascending aorta. This finding would support the thought that hemodynamic stress greatly affects the development of BAV-associated aortopathy. Biological pathways involved in smooth muscle cell contractile phenotype, metabolism, and cell stress showed decreased expression in BAV vs. TAV [85].

On the other hand, a metabolomic study on calcific aortic valve leaflets showed no significant differences between the lipid profiles of BAV and TAV patients, suggesting that underlying biochemical activity and the state of cells/tissues were similar between TAV and BAV during the calcific aortic valve disease [86]. This result is in line with the finding that the overall calcific BAV and calcific TAV gene expression profiles in aortic valve tissue were essentially identical, suggesting that the mechanisms leading to BAV formation are independent of those leading to BAV calcification [87]. However, Surendran et al. found also that elevated lysophosphatidic acid (the end product of lipoprotein (a) metabolism) levels in plasma samples could have the potential to select patients who have a greater need for valve replacement [86].

Another study discovered that there is a predisposition to senescence of smooth muscle cells of the dilated ascending aorta in BAV patients shown as accumulation of unrepaired double-strand DNA breaks. Even more, these cells have a collagen-depletion profile maintained by the activation of p38 protein kinase [88].

## 7. Is There a Biomarker for Bicuspid Aortopathy?

There is a growing interest in the medical community to find biomarkers in BAV [89]. Remember that a biomarker is a measurable biological molecule that is found in the blood and other fluids or tissues of the body, and is a sign that allows evaluating the process of a disease. To date, no specific biomarker of bicuspid aortopathy is used in daily clinical practice and current data are limited, mainly focusing on alterations of the aorta [90,91]. To make decisions about the optimal time for surgery, establish risk profiles, assess the patient’s prognosis, and monitor the progression of aortic calcification or dilation of the aortic wall, etc., it would be extremely important to have reliable biomarkers. We also know that guiding the time of surgery for aortic dilation only based on image measurement is an understatement, since a high percentage of spontaneous dissections occur below the diameter established as both high risk and indicator of surgery [92].

Diverse studies are focusing their attention on the determination of biomarkers, and several potential candidates related to BAV aortopathy have been already described (Table 3).

Currently, it is known that patients with BAV and a higher degree of severity of stenosis or regurgitation have higher levels of N-terminal pro B type natriuretic peptide (NT-proBNP) compared to the low-grade ones, and those with severe aortic regurgitation have higher levels of troponin-T (measured as high-sensitivity troponin T (hsTnT) [90]. However, these two biomarkers are not specific for bicuspid aortopathy but rather reflect the impact of severe valvular disease in ventricular malfunction.

Other plasma biomarkers of BAV aortopathy, such as matrix metalloproteinases (MMPs) and/or tissue inhibitors of metalloproteinases (TIMPs) in BAV patients [93,94] or the existing association between plasma levels of asymmetric dimethylarginine (ADMA), a competitive endogenous inhibitor of NOS, and the diameter of the ascending aorta in patients with aortic aneurysm, have been proposed [95,96]. In addition, an increase in the soluble receptor for advanced glycation end-products (sRAGE) has been identified in plasma of BAV patients with independence of the dilation of the ascending aorta [97].

Likewise, a possible more specific biomarker has been evaluated: transforming growth factor-β1 (TGF-β1). We know that the TGF-β pathway plays a crucial role in vascular remodeling. Recent studies found elevated serum levels of this molecule in patients with dilated aorta in a context of a syndrome (Marfan syndrome, Loeys-Dietz syndrome) although the results were based on small cohorts (9–30 patients) [102,103]. In 2017, Forte et al. published a study in a small series suggesting that during the 3-year follow-up, a high TGF-β1/sENG (circulating TGF-β1 to its soluble co-receptor Endoglin) ratio is associated with a damaging pattern with high progression towards aortic dilation [98]. However, in other later studies, no association was found between serum levels of TGF-β1 and aortic diameter in BAV patients [90,104]. Contradictorily, other studies presented a group of BAV patients in whom TGF-β1 levels were decreased [90]. One thing to consider is the variability of the reference levels of TGF-β1 in serum according to the study, which compromises the interpretation of results [105]. In order to properly compare studies, the biomarker should be measured with the same laboratory technique and reference levels should be established based on large cohorts of healthy patients.

A further study identified several circulating molecules related to the morphology of the aortic valve and the diameter of the ascending aorta. Specifically, they determined that BAV patients had elevated circulating levels of C-reactive protein (CRP) and endothelial microparticles (EMPs) and decreased levels of alpha-tocopherol as well as its plasma carrier high-density lipoprotein (HDL), measured as apolipoprotein A1 (ApoA1) [99].

One more study was centered at a member of the adipokine family, the apelin peptide, known to be implicated in cardiovascular functions through the release of NO. More importantly, animal models of aortic dilation had been shown to reduce the diameter of the dilated aorta after administration of apelin [106]. Accordingly, Şimşek et al. found that serum apelin was decreased in the BAV patients with dilation of ascending aorta compared to the BAV patients without aortic dilation [101]. Thus, apelin could be a serum biomarker for dilation of ascending aorta, but only in the BAV patients.

It is also important to highlight the metabolomic study carried out by Xiong et al. in plasma samples, in which it was found that BAV patients presented an altered arginine and proline metabolism compared to TAV ones. In addition, there was a distortion in the arachidonic acid metabolism pathway, with an increase in these metabolites in BAV patients who suffered from worse baseline hemodynamic stress [100].

Ongoing investigations are focused on locating additional circulating molecules, easily measurable in the laboratory, related to intracellular pathways, not only proteins but also small circulating ncRNAs.

Definitely, at the moment, there are no solid data that allow the use of biomarkers of bicuspid aortopathy in clinical practice and more research and resources are needed in this field.

## 8. Future Directions

The search for the ultimate genetic or epigenetic cause of the different bicuspid phenotypes should be facilitated by the next-generation sequencing tools that allow study of large populations at low cost.

In the near future, studies should also be directed to improve diagnostic and stratification criteria to better predict a more precise personalized risk for BAV patients. To achieve this proposal, imaging methods around the use of modern techniques, such as CMR-4D, are essential [107].

The novel field of the ncRNAs requires further studies to fully understand how they are regulated and the molecular mechanisms through which they carry out their action. Uncovering the significance of ncRNAs in the origin of the BAV aortopathy would not only allow a path to find biomarkers for earlier diagnosis, prior to the appearance of symptoms, but also contribute to the development of new RNA-based disease therapies [68].

Additional omic studies in plasma will be essential for the identification of more precise biomarkers that could be used in clinical practice. In addition, basic research with cell and/or animal models should continue in the direction of clarifying the molecular pathways that lead to valve degeneration in order to identify therapeutic targets that are absolutely lacking.

## 9. Conclusions

**Figure 3:**
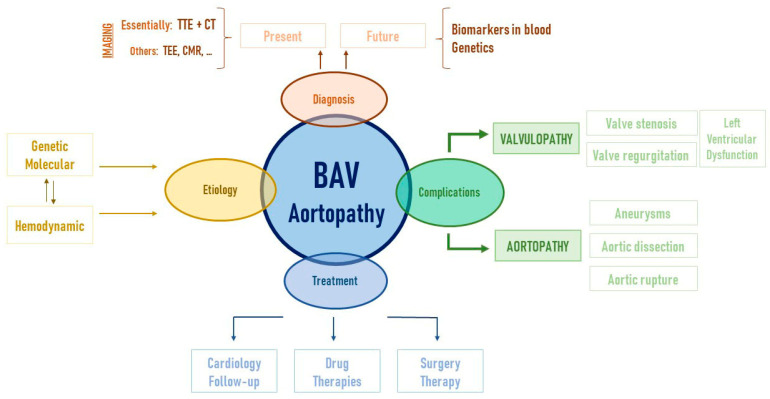
Bicuspid aortopathy is a complex syndrome that includes many parts. Translational research is essential, from the laboratory to daily medical practice, in order to address not only each piece (etiology, diagnostic methods, complications, and treatment) but also the whole problem. In this way, each step that improves scientific knowledge in bicuspid aortopathy improves the lives of the patients who suffer from it. TTE: transthoracic echocardiography; CT: computed tomography; TEE: transesophageal echocardiography; CMR: cardiac magnetic resonance.

Biomedical research on BAV has only grown in recent years. However, it seems that despite the many pieces of the puzzle being obtained, we are only looking at a draft of the final drawing (Figure 3). Single gene mutations have been shown not to explain most cases of non-syndromic BAV. Phenotypic and genotypic heterogeneity is a fact. Knowledge about genetics, hemodynamic impact on tissues, cellular and molecular biology, signaling pathways and possible biomarkers has increased, bringing great advances. However, one of the biggest problems in this field of research remains the unification and interconnection of the findings. Linking discoveries and alterations associated with BAV is a current challenge.

Another major challenge is finding biomarkers for the early development of the disease. As we know, the presence of a BAV is a congenital defect that predisposes to the development of valve and aortic disease. In general, the disease is diagnosed in late stages, when the valve or aortic problem is already established, having gone unnoticed until then. In the future, the ongoing struggle for a thorough understanding of the mechanisms that cause the disease will give the possibility of detecting alterations in earlier. New biomarkers, along with targeted therapies, can change the clinical course of bicuspid aortopathy.

Despite advances, the true pathogenesis leading to bicuspid aortopathy disease is not fully understood. In the coming years, we will see great advances in this field, thanks to the fervent research that continues to be developed.

## Figures and Tables

**Table 1:** Main genes associated with bicuspid aortopathy.

Gene	OMIM	Pathway/Role	Mutation Consequence	Reference
*NOTCH1*	190198	Development of valve and endocardial cushions	Defect on the aortic valve and de-repression of calcium deposition	[33,34]
*GATA4*	600576	Cardiovascular embryogenesis	Enhanced susceptibility to BAV	[35,36]
*GATA5*	611496	Cardiovascular embryogenesis	BAV development	[36,37]
*GATA6*	601656	Cardiovascular embryogenesis	Enhanced susceptibility to familial BAV	[36,38,39]
*TBX5*	601620	Cardiac development	Atrial fibrillation. Segregates with BAV	[40]
*SMAD6*	602931	TGF-β signaling pathway	Related with BAV + aortic aneurysm	[41,42]
*FBN1*	134797	Integrity of the aortic media	Related with isolated BAV and Marfan syndrome with aortic root dilation	[43]
*ROBO4*	607528	Transmembrane receptor	Segregating with ascending aortic aneurysm in BAV	[44]
*ACTA2*	102620	Smooth muscle α-actin	Related with familial ascending aortic aneurysm in BAV	[45]
*ELN*	130160	Arterial development	Correlated with ascending aorta diameter in BAV	[46]

**Table 2:** MicroRNAs with differential expression in BAV patients or BAV-associated aortopathies.

miRNAs	Regulation	Target	BAV-Aortopathy	Reference
34 out of 1583	8↑ 26↓	−	BAV	[67]
miR-141	↓	BMP2-dependent calcification-related genes	BAV + aortic stenosis	[67]
miR-424	↓	SMAD7	BAV + aortic aneurysm	[68]
miR-16, miR-26a, miR-27a miR-30b, miR-130, miR-195, miR-497	↓	−	BAV	[69]
miR-130, miR-195, miR-497	↓	Pro calcifying genes ALPL and BMP2	BAV + aortic stenosis/aortic regurgitation	[69]
miR-133a miR-143	↓	TIMP2	BAV + aortic aneurysm	[70]
miR-122 miR-486	↓	TGF-β pathway-related genes	BAV	[71]
miR-130a	↑	TGF-β pathway-related genes	BAV	[71]
miR-718	↓	Blood vessel remodeling and focal adhesion pathways-related genes	BAV + aortic aneurysm	[71]

Arrow up: upregulation. Arrow down: downregulation.

**Table 3:** Potential biomarkers for the early diagnosis of BAV or its associated aortopathies.

Biomarker	Levels	BAV Associated-Aortopathy	Reference
MMPs/TIMPs	↓TIMPs	BAV + aortic aneurysm	[93]
	↑MMPs	Levels of MMP-2 correlate with aortic dimensions in BAV + aortic aneurysm	[94]
ADMA	↑	Non-stenotic BAV + aortic aneurysm	[95]
	↑	Aortic aneurysm independently of valve morphology	[96]
sRAGE	−	Levels in plasma indicative of BAV status	[97]
NT-ProBNP hsTnT hsCRP	↑ ↑ ↑	BAV + aortic stenosis and regurgitation BAV + aortic regurgitation BAV without any correlation with aortic diameter.	[90]
TGF-β	↑	Higher serum TGF-β/sENG ratio in BAV patients undergoing valve replacement due to aortic stenosis	[98]
	↓	BAV independently of aortic dilation	[90]
Alpha-tocopherol CRP, EMPs	↑CRP, EMPs ↓Alpha-tocopherol	BAV	[99]
Pro/Arg pathways	↓	BAV + aortic stenosis	[100]
Apelin	↓	BAV + aortic aneurysm compared to BAV without ascending aorta dilation and control subjects	[101]

Arrow up: upregulation. Arrow down: downregulation.
